# Efficient Synthesis of 2D Mica Nanosheets by Solvothermal and Microwave-Assisted Techniques for CO_2_ Capture Applications

**DOI:** 10.3390/ma16072921

**Published:** 2023-04-06

**Authors:** P. Vishakha T. Weerasinghe, Shunnian Wu, W. P. Cathie Lee, Ming Lin, Franklin Anariba, Xu Li, Debbie Hwee Leng Seng, Jia Yu Sim, Ping Wu

**Affiliations:** 1 Entropic Interface Group, Engineering Product Development, Singapore University of Technology and Design, 8 Somapah Road, Singapore 487372, Singapore; puwakdandawe@mymail.sutd.edu.sg (P.V.T.W.); shunnian_wu@sutd.edu.sg (S.W.); cathie_lee@sutd.edu.sg (W.P.C.L.); franklin_anariba@sutd.edu.sg (F.A.); 2 Institute of Materials Research and Engineering, Agency for Science, Technology and Research (A*STAR), Singapore 138634, Singapore; m-lin@imre.a-star.edu.sg (M.L.); x-li@imre.a-star.edu.sg (X.L.); debbie-seng@imre.a-star.edu.sg (D.H.L.S.); 3 Anariba Brands Group, Science, Mathematics and Technology (SMT), Engineering Product Development (EPD), Singapore University of Technology and Design, Singapore 487372, Singapore; 4 Institute of Sustainability for Chemicals, Energy and Environment, Agency for Science, Technology and Research (A*STAR), Singapore 627833, Singapore; jia_yu_sim@isce2.a-star.edu.sg

**Keywords:** mica, 2D, solvothermal, microwave, CO_2_ capture

## Abstract

Mica, a commonly occurring mineral, has significant potential for various applications due to its unique structure and properties. However, due to its non-Van Der Waals bonded structure, it is difficult to exfoliate mica into ultrathin nanosheets. In this work, we report a rapid solvothermal microwave synthesis of 2D mica with short reaction time and energy conservation. The resulting exfoliated 2D mica nanosheets (eMica nanosheets) were characterized by various techniques, and their ability to capture CO_2_ was tested by thermogravimetric analysis (TGA). Our results showed an 87% increase in CO_2_ adsorption capacity with eMica nanosheets compared to conventional mica. Further characterization by Fourier-transform infrared spectroscopy (FTIR) and X-ray photoelectron spectroscopy (XPS), as well as first-principles calculations, showed that the high specific surface area and deposited K_2_CO_3_ layer contribute to the increased CO_2_ adsorption on the mica nanosheets. These results speak to the potential of high-quality eMica nanosheets and efficient synthesis processes to open new avenues for new physical properties of 2D materials and the development of CO_2_ capture technologies.

## 1. Introduction

The discovery of graphene sparked worldwide interest in the research and development of 2D materials [1], such as boron nitrides, metal di chalcogenides, phosphorene and many others [2,3,4,5]. Among these, 2D eMica nanosheets (exfoliated mica nanosheets) have emerged as an interesting 2D nanomaterial that finds use in a variety of applications, such as electron tunnelling devices [6], anti-corrosive barriers [7], and flexible and transparent devices [8].

Mica is a naturally occurring mineral that crystalizes into sheet-like alumina silicate layers bonded by ionic bonds. In this particular instance, we are referring to muscovite mica whose chemical structure is represented by the formula KAl_2_(Si_3_Al)O_10_(OH)_2_. This mineral consists of three layers, with an octahedral layer of alumina sandwiched between two identical tetrahedral layers of silica. These layers are negatively charged and separated from each other, connected by potassium cations located in the interlayers. As a result, there is a strong Coulombic interaction between adjacent layers [9]. Due to this complex structure, it is challenging to separate mica into single or few layers. Therefore, bulk mica is a cheap and commercial raw material, while nanosheet mica is extremely rare and expensive [8]. Hence, the scalable fabrication of high-quality eMica nanosheets from natural mica using a facile and efficient method would be a significant contribution to science and technology, both fundamentally and practically, as it could unlock new properties and new applications for this traditional material.

The combustion of fossil fuels has led to an increase in atmospheric CO_2_ levels and concurrent global warming. This accumulation of gas in the atmosphere has also led to adverse effects, such as air pollution and abrupt weather patterns [10]. To mitigate these negative consequences, it is necessary to explore effective methods of capturing and storing CO_2_ from ambient air. As such, research groups have shown great interest in using sorbents for carbon capture. Both liquid- and solid-phase materials are utilized for the adsorption of CO_2_. Nonetheless, solid adsorbents are preferred over liquid sorbents due to their ability to overcome limitations, such as equipment corrosion, solvent loss, and high energy requirements for regeneration [11,12]. As solid sorbents, numerous porous materials have been examined as potential molecular sieves for capturing CO_2_. For instance, research indicates that graphene organic frameworks (GOFs), metal organic frameworks (MOFs), 2D layer double hydroxides (LDHs) and 2D transition metal dichalcogenides (TMDs) possess 3D ordered structures with narrow and uniform pore size distributions that effectively retain CO_2_ molecules [13,14,15,16,17]. However, these structures have drawbacks, such as comparatively low thermal stability, which is an important property in regeneration, and the high cost associated with large-scale preparation of these materials. Therefore, clay minerals have emerged as a viable option due to their exceptional performance in adsorption and catalysis [18]. Their widespread availability, stability, cation framework, and electrochemical charge storage provide a powerful platform for carbon capture. Therefore, in this study we investigate CO_2_ capture and the adsorption mechanism of 2D eMica nanosheets with high specific surface area, uneven electron distribution and high number of active sites on the surface compared to bulk mica [19].

Previous studies have reported the chemical exfoliation of mica through a process involving the dissolution of potassium hydroxide (KOH) + THF (tetrahydrofuran) solution, autoclaving at 250 °C for 72 h, followed by microwave reaction for 5–8 min at 60 Hz 1000 W [20]. However, this method requires a long reaction time and high energy consumption. To address these issues, we introduced a combined microwave-assisted solvothermal method using KOH as an intercalant and N, N-Dimethylformamide (DMF) as a polar solvent. This method supports the microwave dielectric heating of reactants by transferring energy to microwave-absorbing polar solvents, along with an increase in self-generated pressure inside the closed reaction vessel. These actions lead to rapid exfoliation and shorten the reaction time. The microwave-treated mica was further expanded and separated by sonication. The synthesized mica nanosheets were thoroughly investigated for their properties. Moreover, the applicability of 2D eMica nanosheets for CO_2_ capturing and its reusability were demonstrated using thermogravimetric analysis (TGA), while the CO_2_ adsorption mechanism was further elucidated by FTIR and XPS analysis and first-principles calculations.

## 2. Materials and Methods

### 2.1. Materials and Reagents

Natural ground mica with 99.5% purity, manufactured using the wet method, was purchased from Huajing Mica Co., Ltd., Shijiazhuang, China. N,N-Dimethylformamide (≥99.8%, A.C.S. R) was purchased from Sigma-Aldrich Pte Ltd. (St. Louis, MO, USA) Potassium hydroxide (KOH) was purchased from GCE^®^, Laboratory Chemicals (Steinhausen, Switzerland).

### 2.2. Synthesis of Exfoliated Mica Nanosheets

To prepare an organic potassium solution, 10 g of KOH was added to 100 mL of N,N-Dimethylformamide (99% DMF) and stirred at room temperature for 48 h. Then, 2 g of natural mica (bulk mica) was added to the solution and stirred at room temperature for 24 h. The sample was heated at 200 °C for 1 h in a microwave oven, following the four phases of the heating profile shown in Figure S1. Subsequently, the organic solvents were removed by centrifugation (14,000 rpm for 30 min). The resulting K-intercalated mica sample was dispersed in 100 mL of 1% HCl solution and sonicated for one hour, with 2 s pauses between 5 s pulses. The sample was washed with deionized water using several centrifugation cycles (14,000 rpm for 30 min) to remove excess ions from KOH and HCl and neutralize the pH. The resulting material was designated as expanded mica and subjected to further characterization. The neutralized expanded mica dispersion was then subjected to centrifugation at a lower speed of 6000 rpm for 10 min to remove bulk particles, and the resulting supernatant was named as exfoliated mica nanosheets (eMica nanosheets). Each sample stage was dried using freeze drying.

### 2.3. Instrumentation and Characterization

Microwave synthesis was performed using the flexiWAVE Advanced Flexible Microwave Synthesis Platform (ITS Science & Medical Pte Ltd., Singapore). Sonication was performed using a Q125 Tip Sonicator with variable power from 0 to 500 W and an oscillation frequency of 20 kHz (Qsonica L.L.C., Newtown, CT, USA). Nanosheet morphology was studied using JSM-7600F scanning electron microscopy (SEM) coupled with an energy dispersive X-ray spectroscopy (EDS) (JEOL, Tokyo, Japan) and Titan 80/300 scanning/transmission electron microscope (TEM) (200 KV) (Thermofisher, Waltham, MA, USA). X-ray photoelectron spectroscopy (XPS) was performed using XPS Theta Probe (Thermo Scientific, Waltham, MA, USA) to determine the chemical state of the elements. The powder diffraction pattern was visualized by X-ray diffraction (XRD) (Bruker D8 DISCOVER, Billerica, MA, USA). Fourier-transform infrared spectroscopy (FTIR) analysis was carried out using a Spectrum 2000 FTIR spectrophotometer (Perkin Elmer, Akron, OH, USA). The FTIR spectra were recorded in attenuated total reflectance (ATR) mode with a resolution of 4 cm^−1^ between 4000 cm^−1^ and 600 cm^−1^, and the sample scan time was set to 64 s. Brunauer-Emmett-Teller analysis (BET) for surface analysis was performed using the ASAP 2020 system (Micrometrics, Norcross, GA, USA). The BET test was performed with 0.1 g powder samples and outgassed at 200 °C. Thermogravimetric analysis (TGA) of the samples for CO_2_ capture of the samples was performed using a TGA Q50 analyzer (TA Instruments, New Castle, DE, USA). Samples were degassed with N_2_ at 150 °C for 1 h prior to testing, and backfilled with CO_2_ at room temperature to obtain the time-dependent weight gain profile.

### 2.4. Computer Simulations

The first-principles calculations were carried out using a periodic supercell model by the Vienna Ab-initio Simulation Package (VASP) [21] with the Perdew-Burke-Ernzerhof (PBE) generalized gradient approximation (GGA) exchange-correlation functional [22]. A projector augmented wave (PAW) method [23,24] was used as a plane wave basis set, and a tested 500 eV kinetic energy cutoff was set for the plane-wave expansion. The contribution of long-range dispersion (van der Waals interaction) based on the DFT+D3 correction method of Grimme et al. [25] was applied to all calculations. For the geometric optimization and energy calculation, self-consistent energy tolerance was set as 1.0 × 10^−6^ eV, and maximum force tolerance on each atom was set to be smaller than 0.01 eV/Å. The smearing value was set as 0.1 eV. A Monkhorst−PackK-points mesh [26] was used for sampling the Brillouin zone, where the number of K-points (N_K_) is changed to keep (N_K_ × L) (L: the lattice constant) equal to ~30 Å and ~50 Å for structural relaxations and electronic calculations, respectively.

The stable supercell structure of mica, mica monolayer and K_2_CO_3_-deposited mica monolayer obtained in our previous work [27] was adopted to investigate their CO_2_ adsorption behavior. Mica surface was obtained by cleaving the bulk in the (001) direction. The adsorbate CO_2_ and half mica structures (including K_2_CO_3_ layer of K_2_CO_3_-deposited mica) were allowed to be relaxed, whereas the remaining half mica structures were to be fixed in their bulk positions. At least 20 Å vacuum is placed on both sides of all surfaces to avoid image interaction in the periodic boundary condition.

The CO_2_ adsorption energy $E_{ad}$ of the mica surface is defined as $E_{ad}=E_{surface/CO_{2}}-E_{CO_{2}}-E_{surface}$ where $E_{surface/CO_{2}}$ is the total energy of CO_2_ plus each surface, $E_{CO_{2}}$ the energy of CO_2_, and $E_{surface}$ the total energy of the surface. Therefore, a lower value of $E_{ad}$ indicates a stronger adsorption of CO_2_ to the surface. The charge in an atom was defined as the difference between the valence charge and the Bader charge. The Bader charge was determined with the Bader scheme of charge density decomposition [28,29,30].

## 3. Results Discussion

### 3.1. Structural and Morphological Characterization

**Figure 1:**
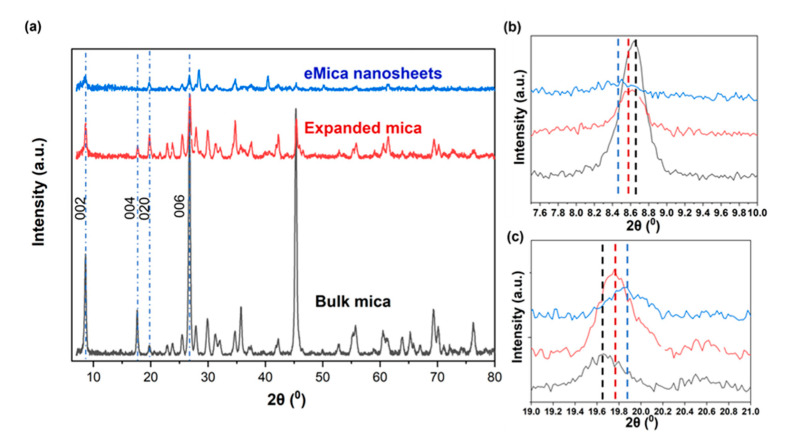
XRD of (**a**) bulk mica, expanded mica and eMica nanosheets, (**b**) enlarged image of peak between 7 and 17, and (**c**) enlarged image of peak between 17 and 26.

The phase of the synthesized eMica nanosheets is confirmed to be a monoclinic potassium aluminum silicate hydroxide (KAl_3_Si_3_O_10_(OH)_2_; muscovite) with space group C2/c (15), indexed by JCPDF card number 01-084-1302. The XRD spectrum (Figure 1a) of the expanded mica indicates relatively lower intensity of the 002 peak, demonstrating the separation of mica into thin sheets with short periodicity due to the expansion of mica by microwaves (spontaneous heat of KOH/DMF solution) and sonication. The peak intensities of the 002 peak further decreased in the eMica nanosheets when the remaining bulk particles were removed by centrifugation at 6000 rpm for 10 min. A similar decrease in the (002) peak due to the reduction in the number of layers was observed in MoS_2_ nanosheets and g-C_3_N_4_ nanosheets [31]. The average thickness of the samples was calculated using the Scherrer equation (Equation (1)).

(1) $$\tau =\frac{K\lambda }{\beta \mathrm{COS}\theta }$$

where τ is mean size of crystalline, K is dimensionless shape factor, λ is the X-ray wavelength, β is the line broadening at half the maximum intensity (FWHM) and θ is the Bragg angle. The calculated particle thickness was ~30, 20, and 6 nm for bulk mica, expanded mica, and eMica nanosheets, respectively.

In particular, the (002) plane (c-axis) shifts to smaller angle 2θ values of 8.63, 8.57, and 8.47 (Figure 1b) for bulk mica, expanded mica, and eMica nanolayers, respectively, while the peak of the (020) plane shifts to larger angle 2θ values of 19.65, 19.75, and 19.88 (Figure 1c) for bulk mica, expanded mica and eMica nanosheets, respectively, indicating that there is an expansion along the [00n] direction and generation of in-plane compressive strain in the mica nanosheets.

The calculated average interlayer spacing of the (002) plane is 10.43 Å for the mica nanosheets, which is larger than that of bulk mica (10.07 Å). For expanded mica (10.28 Å), this value ranges between those for bulk mica and eMica nanosheets, suggesting that the sample contains a mixture of exfoliated and non-exfoliated mica. The observed increase in the (002) plane spacing causes uniaxial tensile strain along the [00n] direction in the mica crystal. On the other hand, the average spacing of the (020) plane has decreased by ~1.2%, from 4.51 Å for bulk mica to 4.46 Å for the eMica nanosheets, indicating compressive strain along [0n0] direction.

**Figure 2:**
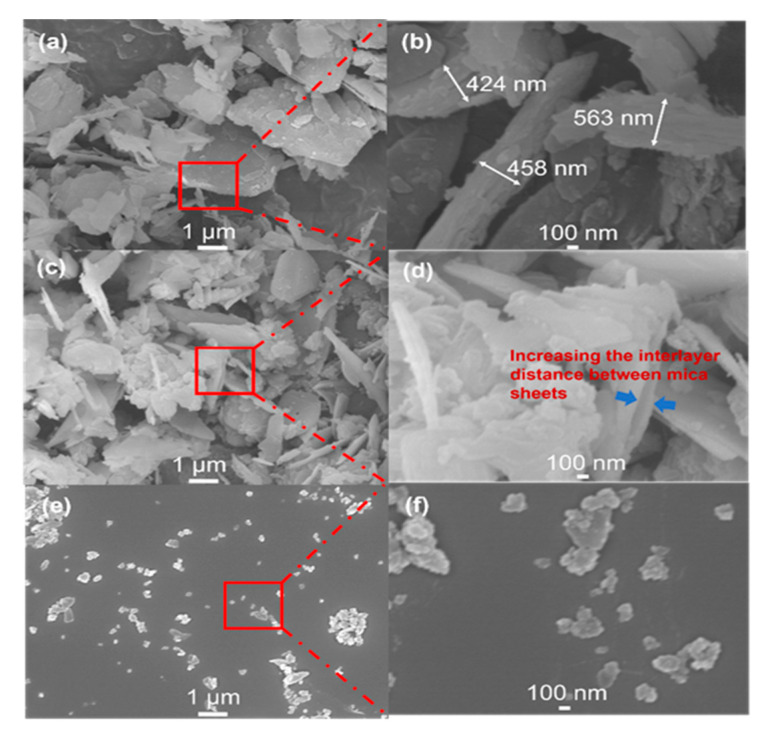
SEM images of (**a**) natural bulk mica and (**b**) its magnification image, (**c**) expanded mica, and (**d**) its magnification image, and (**e**) eMica nanosheets, and (**f**) its magnification image.

**Figure 3:**
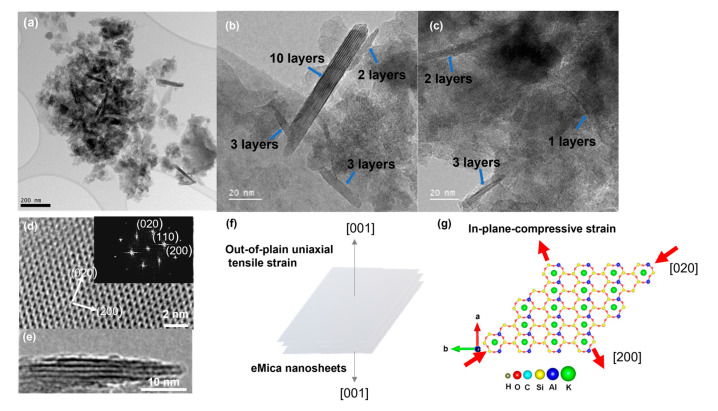
TEM images of eMica nanosheets at (**a**) low magnification, (**b**) moderate magnification, (**c**) high magnification and the view of the edge showing number of layers of nanosheets. HRTEM images of the (**d**) basal plane view of eMica nanosheets and the corresponding fast Fourier transform (FFT) image. (**e**) The cross-section HRTEM is along [001]. Diagram of (**f**) outer plane uniaxial tensile strain and (**g**) in-plane biaxial tensile and compressive strain in eMica nanosheets.

The lateral size and thickness of the mica samples were measured using SEM and TEM. SEM analysis of natural ground mica revealed a morphology constructed by overlapping a set of parallel layers. The dimensions of these layers are irregular, ranging from 0.5 to 2.5 μm in width and several hundred nanometers in thickness (400–500 nm) (Figure 2a,b). In expanded mica, an increase in the distance between the layers is generally observed (Figure 2c,d). Compared to the bulk material, eMica nanosheets exhibited a more uniform lateral size in the range of 100–300 nm in width, (Figure 2e,f) and thickness in the range of 1–10 nm (Figure 3a–c) compared to the bulk. TEM images (Figure 3a) display the edge side of the sheets, which are generally rolled and partially folded due to the high surface tension required for the material to conserve its planarity over a long distance. It is common to observe that these nanosheets contain between 2 to 10 layers, as seen in Figure 3b. The thickness of a layer is measured to be about 1 nm (Figure 3c).

The high-resolution TEM (HRTEM) images of the basal plane of eMica nanosheets and cross-section of particles are shown in Figure 3d and Figure 3e, respectively. The ordered atomic arrangement in Figure 3d exhibits that the eMica nanosheets are of high quality and with less defects. The fast Fourier transformed (FFT) image was obtained and lattice spots can be indexed to the (200) and (020) planes, confirming the corresponding zone axis along the [001] direction. The average measured interlayer spacing is 10.57 Å in the eMica nanosheets, which is about 7% larger than the bulk interlayer spacing (10.07 Å), indicating out-of-plane uniaxial tensile strain (Figure 3f), consistent with the uniaxial tensile strain along the *z*-axis determined by the XRD results.

In the planar orientation, lattice fringes with interplanar spacings of 2.65 Å, 4.43 Å, and 4.71 Å corresponding to the (200), (020), and (110) planes of the ditrigonal are revealed (Figure 3d, FFT image). The labelled (020) atomic plane spacings of the eMica nanosheets are ~1.8% smaller than the corresponding lattice spacings in bulk mica, consistent with the XRD results, indicating biaxial compressive strain (Figure 3g) in the basal plane along (020) and (200).

**Figure 4:**
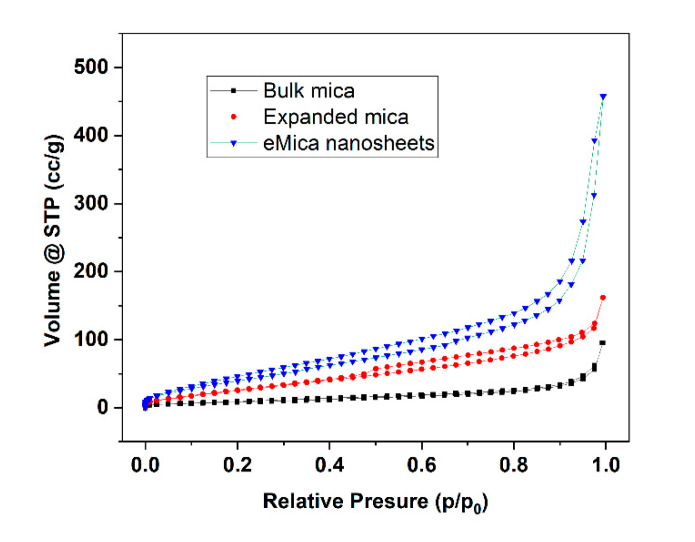
N_2_ Adsorption/desorption isotherms of bulk mica, expanded mica and eMica nanosheets at 77 K.

Specific surface area and porosity were measured using the BET method at Autosorb iQ Station 1, with nitrogen adsorption analyses at 77.3 K (Figure 4). According to the size distribution curves calculated by the BJH method, the specific surface area was significantly increased from 29.1 m^2^/g for bulk mica to 171.3 m^2^/g for eMica nanosheets. Similarly, the pore volume follows the sequence of bulk mica < expanded mica < eMica nanosheet. Table 1 shows the surface area and pore volume of all mica samples.

### 3.2. CO_2_ Capture and Regeneration

**Figure 5:**
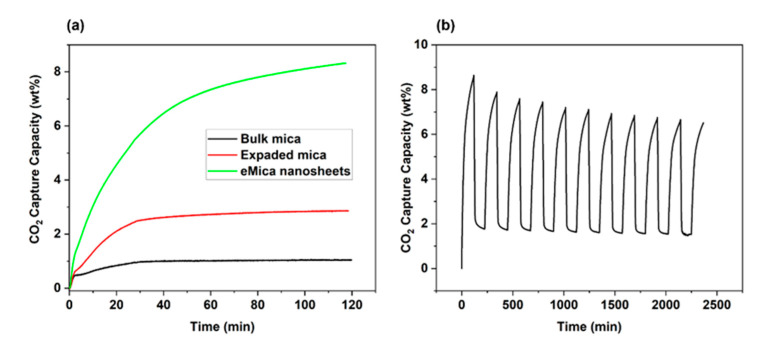
(**a**) CO_2_ adsorption isotherm at room temperature and (**b**) cyclic isotherm of adsorption and desorption.

The application of eMica nanosheets for CO_2_ capture was demonstrated by TGA analysis at room temperature (Figure 5). To remove any adsorbed gases and water vapor prior to purging with CO_2_ at room temperature, all samples were degassed at 150 °C for 60 min. It shows that the adsorption capacity of eMica nanosheets is 87% higher than that of bulk mica. The CO_2_ adsorption capacity increased within 90 min at 30 °C from 1.0 ± 0.02 wt.% for bulk mica to 3.2 ± 0.37 wt.% for expanded mica and to 7.6 ± 1.13 wt.% for eMica nanosheets (Figure 5a). The comparison presented in Table 2 with the literature indicates that the adsorption capability of eMica nanosheets is inferior to that of MOF and GOF, but superior to some other clay minerals which have been functionalized.

In addition, the stability of CO_2_ adsorption capacity was investigated by a cyclic adsorption/desorption test; the result is shown in Figure 5b. In TGA, the sample is subjected to a controlled temperature program in reactive atmosphere (100% CO_2_ atmosphere), and the weight change of the sample is continuously monitored as a function of temperature or time. By measuring the weight change of the sample during CO_2_ adsorption and desorption, the amounts of CO_2_ adsorbed and desorbed were determined. CO_2_ adsorption takes place at room temperature, and the temperature is increased up to 150 °C in the 100% CO_2_ atmosphere to facilitate desorption. When the temperature is increased, the thermal energy of the system also increases, causing the weak bonds between the CO_2_ and the mica surface to break. As a result, the adsorbed CO_2_ may start to desorb or detach from the surface of the mica mineral and return to the gas phase [32]. From the 1st cycle to the 2nd cycle, the desorption performance decreased 1.8%. This could be due to the cavitation collapse caused by the CO_2_ gas flow, which implies the CO_2_ cannot be completely removed by heating to 150 °C alone. At the 5th and 10th cycles, the total adsorption drop was 0.5% and 1%, respectively. The adsorption capacity of eMica nanosheets may decrease more in the first few cycles due to the partial blockage of pores by the adsorbed CO_2_ molecules or impurities. The surface becomes saturated with CO_2_ molecules and the adsorption capacity decreases the adsorption and desorption cycles, causing some changes in the structure of the eMica nanosheets, such as the formation of cracks or the detachment of some layers [33,34,35]. However, after several cycles, the pores, surface, and structure may become more uniform and stable, leading to a slower decrease in adsorption capacity. From this result, it can be concluded that the eMica nanosheet adsorbents have good adsorption capacity, recoverability and stability.

**Table 2:** Comparison of CO_2_ adsorption capacity.

	Adsorption Capacity (wt.%)	Adsorption Conditions
Kaolinite [36]	0.3	25 °C, 1 bar
Bentonite [37]	0.6	25 °C, 1 bar
Amine grafted Zeolite-Y [38]	5	25 °C, 1 bar
Pillared clays (PILCs) from Montmorillonite	5.2	25 °C, 1 bar
eMica nanosheets (This study)	7.6	30 °C, 1 bar
MOF (Cu-BTTri) [39]	14.0	25 °C, 1 bar
GOF (Tannic acid–iron-coordinated compound-derived porous carbons) [40]	14.9	25 °C, 1 bar

Previous studies suggest that CO_2_ sorption by mica or clay surfaces is due to the formation of carbonate complexes as a result of the reaction of CO_2_ with hydroxyl groups of clay or H_2_O [41,42]. P. Giesting et al. [43] have described that CO_2_ reacts with H_2_O present in montmorillonite to form carbonate species such as H_2_CO_3_ and HCO_3_^−^ on montmorillonite samples [43]. These carbonate species may interact with the neighboring basal oxygen planes through hydrogen bonding and electrostatic attraction to the Na cations in the interlayers. Similarly, the mechanism of CO_2_ adsorption from mica could be based on the reaction of the H_2_O with CO_2_, leading to the formation of carbonate species that can attract to the K^+^ interlayers through electrostatic attraction. Nevertheless, CO_2_ physisorption may be a prominent quadrupole interaction between the alkali metal cations and the CO_2_ molecules.

**Figure 6:**
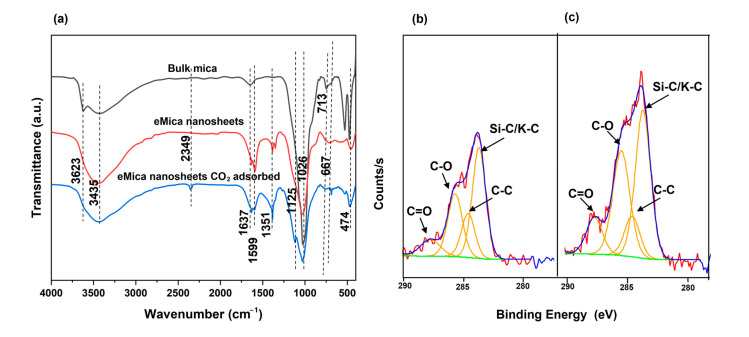
(**a**) FTIR of eMica nanosheets and eMica nanosheets after CO_2_ adsorption. XPS analysis of (**b**) eMica nanosheets and (**c**) CO_2_-adsorbed eMica nanosheets.

To further investigate the CO_2_ adsorption mechanism, FTIR analysis (Figure 6a) was performed for eMica nanosheets and CO_2_-adsorbed eMica nanosheets. CO_2_-adsorbed eMica nanosheets were obtained after the CO_2_ adsorption test in TGA. CO_2_ adsorbed eMica nanosheets were exposed to a 100% CO_2_ environment at room tempereture. For all samples, the characteristic peaks at 1026 cm^−1^ and 713 cm^−1^ are due to Si-O-Si and Al-O-Al [44]. The peaks at 3435 and 1637 are due to Si- or H-bonded OH and structural hydroxyl groups or physiosorbed water, respectively [45,46,47,48]. The ratio between the peak intensities of each of these peaks and Si-O-Si is higher in the eMica nanosheets than in bulk mica. This could be due to the larger specific surface area of the nanosheets compared to bulk mica, which can accommodate a larger number of OH groups and H_2_O-adsorbed active sites. As a result, there is a higher affinity for chemisorption of CO_2_ and formation of carbonate spices by eMica nanosheets compared to bulk mica. The intensity of the peak at 3623 is attributed to the isolated OH. There is a slight decrease in the peak intensity of the OH groups of CO_2_-adsorbed mica due to degassing at 150 °C by N_2_ before CO_2_ purging. The CO_3_^2−^ peak appears at 1351 cm^−1^ in eMica nanosheets and peak intensity increased only slightly after CO_2_ adsorption. Moreover, a CO_2_(ⱱ3) peak can be observed at 2349 cm^−1^, which we attribute to physiosorbed CO_2_.

To further investigate the presences of C species, XPS of eMica nanosheets and CO_2_-adsorbed eMica nanosheets were observed. The peaks at 283.7, 284.6, 285.7–285.5, and 287.7–287.8 eV can be assigned to Si/K-C, C-C, C-O, and C=O, respectively (Figure 6b,c) [49]. Previous studies have shown that potassium carbonate crystallites can be present on air-cleaved mica surfaces [50,51]. This could be the reason why the C=O and C-O peaks in the XPS and CO_3_^2−^ peak in FTIR are visible of the eMica nanosheet samples even before the CO_2_ adsorption test. After subjecting the samples to a 100% CO_2_ atmosphere in TGA, the relative atomic percentages of C-O and C=O increased from 26.4% to 33.8% and from 9.8% to 11.3%, respectively. Meanwhile, the relative atomic percentage of C-C decreased from 17.8% to 11.8% when comparing eMica nanosheets to CO_2_-adsorbed eMica nanosheets. The C=O peaks could have originated from either physiosorbed CO_2_ molecules or chemisorbed CO_3_^2−^ or HCO_3_^−^ species. The higher amounts of desorption by heating to 150 °C during cyclic isotherm and the absence of high intensity increase of carbonate peaks after CO_2_ adsorption in FTIR indicate that physisorption dominates, and chemisorption is secondary on the nanosheet surfaces.

**Figure 7:**
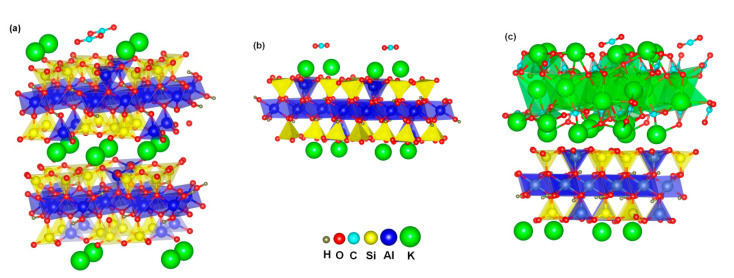
Configuration of adsorbed CO_2_ on (**a**) bulk mica, (**b**) a mica monolayer and (**c**) K_2_CO_3_ deposited mica monolayer.

To understand physical adsorption of CO_2_ on both mica and the mica monolayer surface, the system was simulated with first-principles calculations and their most stable configurations are shown in Figure 7. The adsorbed CO_2_ molecules are at the top of the bridge position of two surface K cations, which may be due to the electrostatic attractive interaction between two O atoms of CO_2_ and two neighboring K atoms.

The calculated CO_2_ adsorption energy values on all surfaces and the corresponding configurational parameters of CO_2_ are summarized in Table 3. It can be seen that mica shows an adsorption energy of −0.39 eV, which is regarded as physisorption since it is below the chemisorption energy threshold of −0.52 eV. The adsorption energy of the monolayer is found to be smaller than that of bulk mica. It is noted that the two O-C bond lengths of adsorbed CO_2_ molecules on the monolayer surface are similar, while they show substantial discrepancy on bulk mica surface. This is consistent with the mechanism that uneven electron distribution in two O atoms of CO_2_ facilitates CO_2_ adsorption [19]. Bader charge analysis indicates a slightly greater charge transfer to CO_2_ from bulk mica than a mica monolayer. The electron transfer is another mechanism to promote CO_2_ adsorption [19], which further contributes to the superior CO_2_ adsorption by bulk mica compared to a mica monolayer.

Our experiments observed significantly improved CO_2_ adsorption by eMica nanosheets in comparison with bulk mica. The calculated CO_2_ adsorption per unit surface area increased by 28.6% between eMica nanosheets and bulk mica. This appears to be consistent with the theoretical simulation of a mica monolayer. However, it is known that preparing mica surfaces that are truly clean is not easy, since mica has a high-energy surface that readily adsorbs water, organic contaminants, and gases from the atmosphere. Previous experimental and theoretical studies suggest the deposition of potassium carbonate (K_2_CO_3_) crystals on the mica surface, [27,51,52,53] which can be attributed to the reaction of ambient water and CO_2_ with surface K^+^ ions. Considering that our eMica nanosheets are prepared and maintained in an open environment rather than under an ultrahigh vacuum, it is relatively impossible to avoid deposition of K_2_CO_3_ on the high-energy mica monolayer surface, which is in agreement with the presence of C=O in XPS analysis of eMica nanosheets (Figure 6c). Therefore, the dramatically increased CO_2_ adsorption by eMica nanosheets may be attributed to the deposited K_2_CO_3_ layers on the surface. Therefore, CO_2_ adsorption by K_2_CO_3_ deposited mica monolayers was simulated, with the most stable configuration shown in Figure 7c and the calculated CO_2_ adsorption energy is shown in Table 3.

The adsorbed CO_2_ molecules are similar in the top of bridge position of two surface K cations on bulk mica and mic monolayer. The CO_2_ adsorption energy of bulk mica is higher than that of the mica monolayer, indicating improved CO_2_ adsorption performance. Theoretical studies reveal that adsorbent surface polarization plays a crucial role in CO_2_ adsorption, while CO_2_ adsorption energy is positively correlated with the <O-C-O angle of adsorbed CO_2_ molecules on studied adsorbents [19]. A positive correlation of CO_2_ adsorption energy with the <O-C-O angle of adsorbed CO_2_ molecules is shown in Table 3, indicating that CO_2_ adsorption depends on the polarization of CO_2_ molecules on mica surfaces. The two O-C bond lengths of adsorbed CO_2_ molecules for a K_2_CO_3_ deposited monolayer show a small discrepancy in comparison with the apparent difference for bulk mica, indicating that uneven electron distribution between two O atoms of CO_2_ is inconsequential to the improvement in CO_2_ adsorption. Table 3 shows that the electron transfer to a CO_2_ molecule from a K_2_CO_3_ deposited monolayer is remarkably increased, indicating its predominant contribution to the observed improvement in CO_2_ adsorption. Therefore, accumulation of electrons on adsorbent surfaces, such as coating compounds with large charge density or doping elements with high valence electrons, to promote larger electron transfer to CO_2_ molecules, appears to be the most effective approach to improve CO_2_ adsorption. However, this K_2_CO_3_ deposition does not continuously cover the entire mica nanosheet surface. AFM examination of air-cleaved mica revealed discontinuous nanosized crystal particles formed on the surface of the air-cleaved mica [51].

**Figure 8:**
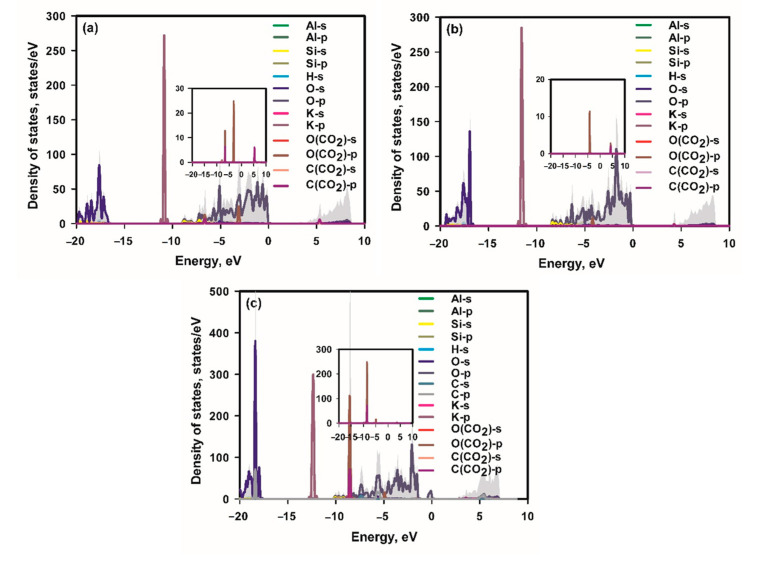
Projected density of states of adsorbed CO_2_ on bulk mica (**a**), a mica monolayer (**b**) and K_2_CO_3_ deposited mica monolayer (**c**). The inset is a magnification of states of C and O of CO_2_.

Figure 8 shows the projected density of states of adsorbed CO_2_ on the studied surface. For the surface of bulk mica, the adsorbed CO_2_ shows hybridization of O-2p and C-2p orbitals at −6.6 eV and 5.3 eV to form the bonds of a CO_2_ molecule. The overlapping of O-2p state of a CO_2_ molecule with the O-2p state of the bulk mica surface at −3.1eV is observed, being in agreement with the electron transfer to CO_2_ from the surface. However, the major K states located at −10.9eV show no hybridization with any states of CO_2_, indicating no bonding with the adsorbed CO_2_ molecules. Therefore, the weak adsorption of bulk mica with CO_2_ is mainly due to the electrostatic attractive interaction. For a mica monolayer surface, the adsorbed CO_2_ shows hybridization of O-2p and C-2p orbitals at 4.3 eV to form the bonds of a CO_2_ molecule. There is overlapping of the O-2p state of a CO_2_ molecule with O-2p states of the surface at −4.2eV, which indicates electron transfer to CO_2_ from the surface, which brings about the adsorption.

Similarly, the main K state located at −11.6 eV shows no overlapping with any states of CO_2_. With regard to a K_2_CO_3_ deposited mica monolayer, the adsorbed CO_2_ shows hybridization of O-2p and C-2p orbitals at −8.4 eV. The overlapping of the O-2p state of a CO_2_ molecule with O-2p states of the surface at −4.9 eV occurs, indicating the existence of electron transfer to CO_2_ from the surface. However, the overlapping of the K state at −12.4 eV with any states of CO_2_ does not occur; therefore, no bonding is formed between CO_2_ and the surface K ions. Such physisorption is advantageous for desorption of CO_2_ for adsorbent regeneration.

## 4. Conclusions

To summarize, we have developed a straightforward and effective solvothermal method that uses microwaves to synthesize eMica nanosheets. These nanosheets have a thickness of less than 10 nm and retain their crystalline muscovite structure. Our experimental results combined with first-principles calculations have demonstrated that the eMica nanosheets exhibit superior CO_2_ adsorption properties compared to bulk mica. The enhanced adsorption is attributed to the high specific surface area of the nanosheets, the porosity between the expanded layers, and the K_2_CO_3_ layer that boosts electron transfer to CO_2_. As a readily available and inexpensive clay mineral on Earth, mica offers great promise for further research in the field of CO_2_ mineralization.

## Figures and Tables

**Table 1:** Values of BET surface area and pore volume.

Sample	Surface Area (m^2^/g)	Pore Volume (cc/g)
Bulk mica	29.1	0.145
Expanded mica	85.7	0.235
eMica nanosheets	171.3	1.022

**Table 3:** CO_2_ adsorption energy ΔE and structural data of adsorbed CO_2_ for studied mica surfaces.

	ΔE (eV)	<O-C-O (deg)	O-C Length (Å)	Accepted Charge |e|
Bulk mica	−0.39	175.64	1.171, 1.183	+0.03
Mica monolayer	−0.30	178.78	1.175, 1.175	+0.01
K_2_CO_3_ deposited mica monolayer	−0.49	170.98	1.178, 1.180	+0.07

## Data Availability

Not applicable.

## Supplementary Materials

The following supporting information can be downloaded at: https://www.mdpi.com/article/10.3390/ma16072921/s1, Figure S1. (**a**) Microwave profile of mica and potassium organic solution, (**b**) Teflon containers of microwave oven and (**c**) microwave oven.
