# Supplementary material for: Efficient Synthesis of 2D Mica Nanosheets by Solvothermal and Microwave-Assisted Techniques for CO2 Capture Applications
Source: Materials (Basel). 2023 Apr 6;16(7):2921. doi: 10.3390/ma16072921 (PMC10096432; doi:10.3390/ma16072921)
Supplement: Supplementary file 1 [file materials-16-02921-s001.zip › materials-2313187-supplementary.pdf]

Supplementary Materials: Efficient Synthesis of 2D Mica Nanosheets by Solvothermal and Microwave-Assisted Techniques for CO_2 Capture Applications

P. Vishakha T. Weerasinghe [1], Shunnian Wu [1], W. P. Cathie Lee [1], Ming Lin [2], Franklin Anariba [1,3], Xu Li [2,4], Debbie Hwee Leng Seng [2], Jia Yu Sim [4] and Ping Wu [1,*]

1 Entropic Interface Group, Engineering Product Development, Singapore University of Technology and Design, 8 Somapah Road, Singapore 487372, Singapore; puwakdandawe@mymail.sutd.edu.sg (P.V.T.W.); shunnian_wu@sutd.edu.sg (S.W.); cathie_lee@sutd.edu.sg (W.P.C.L.); franklin_anariba@sutd.edu.sg (F.A.)
2 Institute of Materials Research and Engineering, Agency for Science, Technology and Research (A*STAR), Singapore 138634, Singapore; m-lin@imre.a-star.edu.sg (M.L.); x-li@imre.a-star.edu.sg (X.L.); debbie-seng@imre.a-star.edu.sg (D.H.L.S.).
3 Anariba Brands Group, Science, Mathematics and Technology (SMT), Engineering Product Development (EPD), Singapore University of Technology and Design, Singapore 487372, Singapore
4 Institute of Sustainability for Chemicals, Energy and Environment, Agency for Science, Technology and Research (A*STAR), Singapore 627833, Singapore; jia_yu_sim@isce2.a-star.edu.sg.
* Correspondence: wuping@sutd.edu.sg; Tel.: +65-94574161

S1. Microwave of mica in potassium organic solution

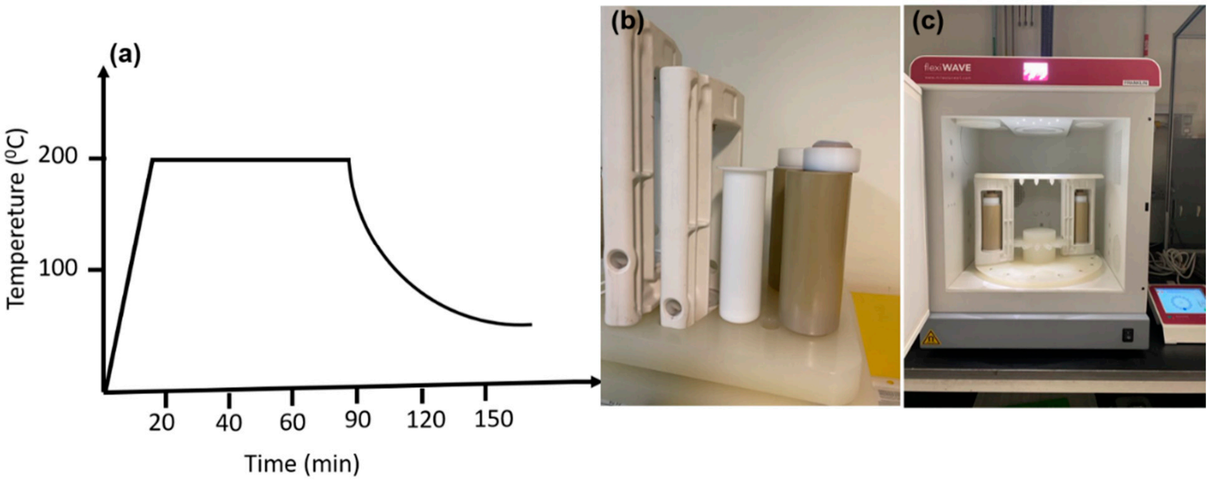

Figure S1: (a) Microwave profile of mica and potassium organic solution, (b) Teflon containers of microwave oven and (c) microwave oven

The sample was heated to 200°C over a period of 20 minutes, with a ramping rate of 10°C per minute. Subsequently, the temperature was maintained at 200°C for one hour in a microwave oven before being allowed to cool down to room temperature. This temperature profile was chosen due to the limitations of closed reaction vessels in terms of pressure and temperature control.
